# Do guidelines for treating chest disease in children use Cochrane Reviews effectively? A systematic review

**DOI:** 10.1136/thoraxjnl-2016-208790

**Published:** 2017-04-26

**Authors:** Andrew P Prayle, Tessy Cox, Sherie J Smith, Joanne Rycroft-Malone, Kim S Thomas, Dyfrig A Hughes, Alan R Smyth

**Affiliations:** 1 Division of Child Health, Obstetrics and Gynaecology, University of Nottingham, Nottingham, UK; 2 School of Healthcare Sciences, Bangor University, Bangor, UK; 3 Centre of Evidence Based Dermatology, University of Nottingham, Nottingham, UK; 4 Centre for Health Economics and Medicines Evaluation, Bangor University, Bangor, UK

**Keywords:** Asthma Guidelines, Paediatric Lung Disaese

## Abstract

Cochrane Reviews summarise best evidence and should inform guidelines. We assessed the use of Cochrane Reviews in the UK guidelines for paediatric respiratory disease. We found 21 guidelines which made 1025 recommendations, of which 96 could be informed by a Cochrane Review. In 38/96 recommendations (40%), some or all of the relevant Cochrane Reviews were not cited. We linked recommendations to 140 Cochrane Reviews. In 37/140 (26%) cases, the guideline recommendation did not fully agree with the Cochrane Review. Guideline developers may fail to use Cochrane Reviews or may make recommendations which are not in line with best evidence.

## Introduction

Clinical practice guidelines support optimal decision making in medical care. Guidelines should use the best available evidence.^1^ Systematic reviews use transparent criteria (such as Grading of Recommendations Assessment, Development and Evaluation (GRADE)) to evaluate the quality of evidence^2^ and so systematic reviews (where available) should be the primary source of evidence in guidelines. The Cochrane Collaboration produces systematic reviews (‘Cochrane Reviews’) using a rigorous methodology. These are peer reviewed at the protocol and review stage, and are updated regularly.^3^ Previous work indicates that guidelines do not make full use of Cochrane Reviews.^4^ ^5^ This represents research wastage, and may lead to suboptimal medical care.

Respiratory disease in children is common—20% of children visiting the emergency department, with a medical problem, will have a respiratory illness.^6^ However, the paediatric respiratory evidence base is limited. Nearly half of children with respiratory disease receive a medication which is off-label or unlicensed.^7^ It is particularly important that guidelines for respiratory disease in children make the best use of this limited evidence. We examined the use of evidence from Cochrane Reviews in guidelines for respiratory disease in children.

We aimed to understand the use of the best available evidence in the field of paediatric respiratory medicine. We systematically examined the use of Cochrane Reviews in the UK clinical guidelines for lower respiratory diseases in children and we examined the agreement between the guideline recommendations and the Cochrane Reviews. We investigated the association between guideline commissioning agency, the topic, the publication year and the use of alternate high-quality evidence on whether Cochrane Reviews were cited, and whether their conclusions were followed.

## Methods

We identified all the respiratory guidelines in the UK for lower respiratory tract disease for children via database and web searches. We simultaneously identified all the Cochrane Reviews relevant to paediatric respiratory medicine, via the Cochrane library. For each guideline, we included all recommendations pertaining to an intervention for lower respiratory tract disease in children. For each recommendation, we identified if there was a Cochrane Review which could inform it, and which had been published at least 1 year prior to the guideline. We mapped each guideline recommendation to relevant Cochrane Reviews.

For each linked guideline recommendation—Cochrane Review, we categorised the agreement between the guideline recommendation and the Cochrane Review into one of four categories: (i) totally, (ii) partially, (iii) not in agreement or (iv) a strong guideline recommendation where the Cochrane Review concluded that there was not enough evidence to draw a conclusion (see online supplementary tables S1 and S2 for definitions and examples). Where guideline recommendations disagreed with the Cochrane Review, we categorised the extent of the disagreement. The protocol (including study eligibility criteria and statistical analysis plan) was produced in advance of the data collection, is available at the University of Nottingham ePrints server and as online supplementary files 2 and 3). Detailed methods are provided in online supplementary information.

10.1136/thoraxjnl-2016-208790.supp1 supplementary data

## Results

### Guidelines and Cochrane Reviews identified

**Figure 1:**
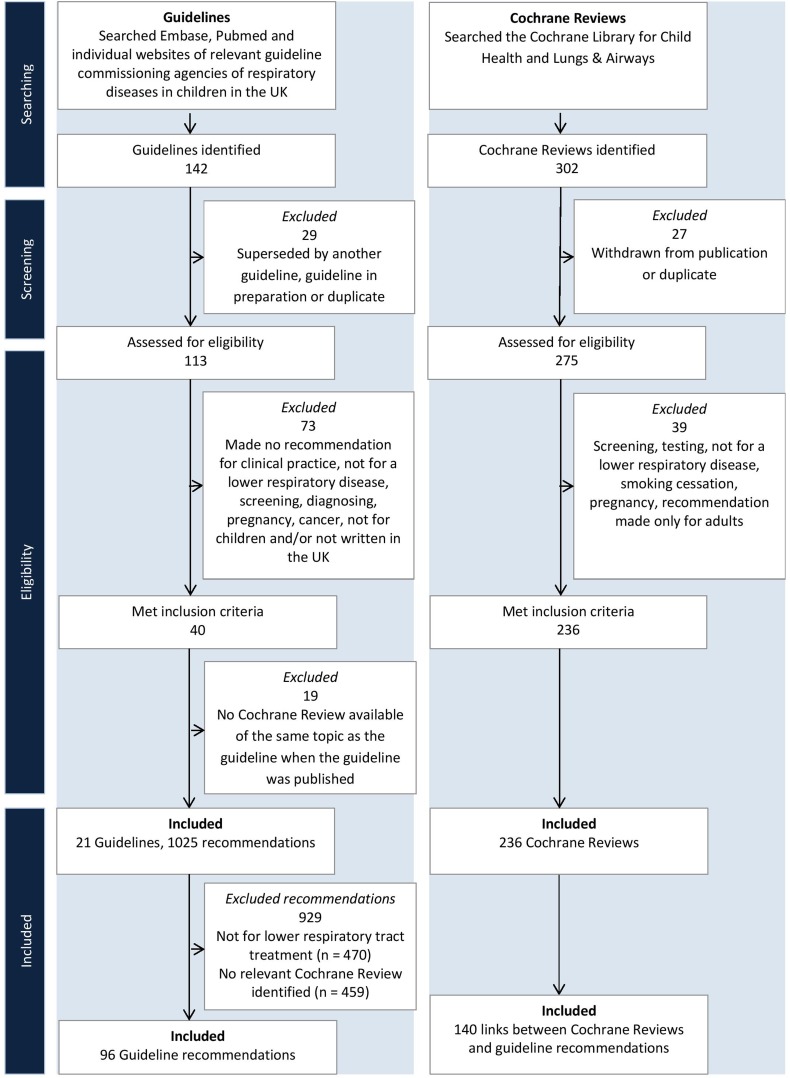
Flow diagram of the selection of guidelines and Cochrane Reviews.

We included 21 guidelines and 236 Cochrane Reviews (see figure 1). The 21 guidelines made 1025 recommendations, of which 555 were for treatment of lower respiratory disease in children. We identified relevant Cochrane Reviews for 96 (17.3%) of these 555 recommendations.

Of the 96 recommendations that could use Cochrane Reviews, 28/96 (29%) did not use any, and 10/96 (10%) did not use all the available Cochrane Reviews. There were 140 instances where a Cochrane Review could be linked to at least one guideline recommendation. Of these, 103/140 (74%) were in agreement, 13/140 (9%) were partially in agreement, 5/140 (4%) disagreed and 19/140 (13%) were strong recommendations but the Cochrane Review did not draw a conclusion. Few Cochrane Reviews in paediatric respiratory medicine were able to draw a strong conclusion, 96/283 (34%).

**Figure 2:**
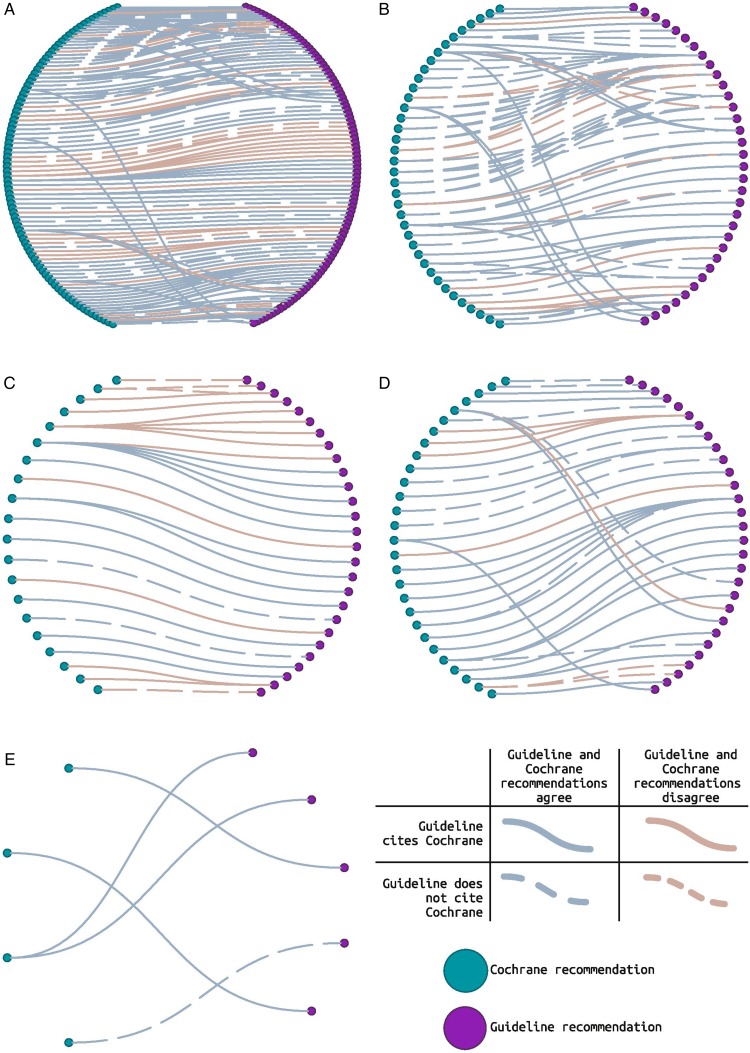
Do Cochrane Reviews influence clinical practice guideline recommendations? Evidence network diagram to show the links between Cochrane Reviews and Guideline recommendations. Each individual guideline recommendation is represented by a purple node, and each Cochrane Review by a green node. A solid blue line connecting a guideline recommendation to a Cochrane recommendation indicates that the guideline cited the Cochrane Review, and the two are in agreement. A broken line indicates that the guideline did not cite the Cochrane Review. A brown line indicates that the Cochrane Review and guideline were not totally in agreement. Panel A shows all the links. Subsequent panels are subgrouped by disease topic: B=asthma, C=cystic fibrosis, D=respiratory infections and E=respiratory aspects of critical care. An interactive web-based version of the evidence network diagram (which loads in all recent major browsers) is available at www.nottingham.ac.uk/~mszap3/interactive_figure.html and allows the reader to explore the underlying data further.

We summarise these data in figure 2. An interactive version of this figure allowing the reader to directly explore the data within a web browser is available online here: https://www.nottingham.ac.uk/~mszap3/interactive_figure.html.

Further analysis is presented in the online supplementary information, including a sensitivity analysis of our judgements in categorising agreement and an analysis investigating the impact of commissioning agency, guideline topic, guideline year and guideline use of alternate high-quality evidence on the use of Cochrane Reviews.

## Discussion

We found that 38/96 (40%) of guideline recommendations did not use all the relevant Cochrane Reviews. The majority of guideline recommendations were in agreement with Cochrane Review recommendations. We present the data as an interactive figure allowing the reader to explore the links between Cochrane Reviews and guideline recommendations.

Our results are broadly in keeping with studies in other fields such as smoking cessation and neonatal medicine which show that guidelines do not make the best use of Cochrane Reviews. Silagy *et al* 4 found four guidelines for smoking cessation (one from the UK). In the UK guideline, 16/22 recommendations could have cited a Cochrane Review but only 8 recommendations did so. Brok *et al* 5 studied the agreement between guidelines and Cochrane Reviews for newborns in Denmark. Compared with our study, they found similar discrepancies between Cochrane Reviews and guideline recommendations—24% were not in agreement (of which 6% partially agreed and 18% disagreed).

Our study is comprehensive, used an a priori protocol and categorisations were conducted independently by two investigators. The study has limitations, including the subjectivity in decisions regarding agreement and disagreement. We expand on this in online supplementary file 1.

## Conclusion

In spite of the work of the Cochrane collaboration, there are still many treatment decisions where there is no systematic review to inform guideline recommendations. However, we have shown that, even where a Cochrane Reviews exists, guideline developers may not make use of it or may make recommendations contrary to the findings of the review. This study demonstrates that only a minority of recommendations in clinical practice guidelines are based on the highest quality evidence. A great deal of money, time and effort goes into creating and updating Cochrane Reviews. Not using such evidence in guidelines constitutes research waste.
