# Supplementary material for: Do guidelines for treating chest disease in children use Cochrane Reviews effectively? A systematic review
Source: Thorax. 2017 Apr 26;73(7):670–3. doi: 10.1136/thoraxjnl-2016-208790 (PMC6035492; doi:10.1136/thoraxjnl-2016-208790)
Supplement: supplementary data [file thoraxjnl-2016-208790supp001.pdf]

Supplementary information file 1.

Methods

Protocol

The protocol for this systematic review (including study eligibility criteria and statistical analysis plan) was produced in advance of the data collection, is available at the University of Nottingham ePrints server (http://eprints.nottingham.ac.uk/id/eprint/3031, http://eprints.nottingham.ac.uk/id/eprint/3032; and are available as supplementary files 2 and 3 to this manuscript).

Search strategy –for guidelines

We searched for clinical guidelines written in the UK for lower respiratory disease (including the lower airways and up to and including anatomical sites of the epiglottis and also including croup) for children (0-18 years old) in Embase, Pubmed and on individual websites of guideline commissioning agencies (search terms used and the list of all websites in supplementary file 2).

Search strategy – for Cochrane Reviews

We searched the Cochrane library for Cochrane Reviews of treatments for lower respiratory tract disease in children. The searches (for both guidelines and Cochrane Reviews) were conducted between September and December 2012.

Inclusion / exclusion – for Guidelines

We included guideline recommendations for clinical practice which were of an intervention for diseases of the lower respiratory tract in children. We excluded recommendations which did not concern interventions (e.g. diagnostic tests), and recommendations about cancer, smoking cessation, pregnancy, expert opinions and recommendations for specialists not directly affecting patients, e.g. hand washing protocols.

Inclusion / exclusion- for Cochrane reviews

For each guideline recommendation, we identified whether there was a relevant Cochrane Review in the Cochrane library. We defined a relevant Cochrane Review as one which was (i) cited by the guideline or (ii) was not cited but reviewed an intervention which was applied to the same target group and could support or contradict the guideline recommendation(s). We excluded Cochrane Reviews where they had not been published at least one year prior to the publication of the guideline, or prior to the date of the literature search undertaken for the guideline, where this date was published within the guideline. After matching the Cochrane Review to the guideline recommendation, if the guideline cited the relevant Cochrane Review anywhere within the guideline, we assumed that the authors of the guideline were aware of the Cochrane Review, and had used it in writing the guideline recommendation in question.

Cochrane review updates

Cochrane reviews are continuously updated documents. However, previous versions of Cochrane Reviews remain available for download from the Cochrane library. Therefore, for each guideline recommendation, we matched with the most recent version of the Cochrane review (published at least one year prior to the guideline). For this reason within our dataset there may be multiple versions of the same Cochrane review, each one linked to guidelines with differing publication dates.

Data extraction

We extracted from the guidelines the topic, publication year, recommendations about interventions and recommendations based on Cochrane Reviews. We also extracted data regarding the commissioning agency, the use of other high quality evidence (such as a meta-analysis, randomised controlled trial or systematic review). Where more than one commissioning agency was involved in the production of a guideline (e.g. the Scottish Intercollegiate Guidelines Network (SIGN) and the British Thoracic Society (BTS) co-produced an asthma guideline) we considered the collaboration as a new entity (i.e. a SIGN-BTS agency). The individual data items for which data were collected are listed in the study protocol.

Analysis

The agreement between the guideline and the Cochrane Review was assigned to one of four categories, (totally in agreement, partially, not in agreement, or a strong guideline but no conclusion in the Cochrane review; definitions shown in table S1). Two investigators (TC and APP) independently assessed Cochrane Reviews for relevance and agreement. Examples of categorizations are shown in table S2. Disagreements were resolved after discussion with a third party (ARS).

Sensitivity Analysis

The classification of different categorizations of agreement and disagreement requires an element of judgement. We therefore undertook a sensitivity analysis in which we examined the impact of differing categorizations of agreement upon our results. We took all the all the pairs which were "partially in agreement", and categorized them as either "not in agreement", or as "totally in agreement". This allowed us to evaluate the effect of having a "partially in agreement" category upon our results.

Alternate sources of high quality evidence

As guidelines may use alternate sources of high quality evidence, we sought to establish if other evidence had been used for each guideline recommendation. We defined alternate high quality evidence broadly as (non-Cochrane) meta-analyses, systematic reviews or randomised controlled trials. We categorized a guideline recommendation as using alternate high quality evidence if the recommendation referenced sources of alternate high quality evidence. The alternate evidence did not need to be specifically referenced in-line within the recommendation, but could be referenced anywhere within the guideline document (we assumed that the authors of that specific recommendation had used all of the references within the guideline).

Statistical analysis

Our primary unit of analysis (i.e. the denominator) was the individual guideline recommendations. We calculated the proportion of guidelines (with 95% confidence intervals) which identified all, some, or none of the relevant Cochrane Reviews. In a series of analyses using logistic regression, we tested whether the commissioning agency, publication year (as a continuous variable) of the guideline, topic of the guideline and the use of other high quality evidence, were associated with of the use of Cochrane Reviews. We used a series of mixed effects models, in which the predictor variable (e.g. commissioning agency) was modelled as a fixed effects term, with a random intercept and slope for each guideline. We then compared the model with and without the fixed effects term using anova, and report the resultant p value for a summary of the overall effect of the predictor variable. We used the **R** packaged **`lme4`** for the mixed effects model, using the model specification in R formula syntax as **`Y ~ X + (1 + X | Guideline)`**, where **X** is the predictor variable (e.g. commissioning agency), and **Y** is a binary response of whether or not the guideline cited all the available Cochrane evidence for that recommendation.

As each guideline recommendation could potentially be linked to multiple Cochrane Reviews, we calculated the proportion of these links in which the Cochrane Review and guideline were in agreement. Analyses were undertaken with R (version 3.2.0). An interactive plot showing the links between Cochrane Reviews and guideline recommendations was designed and implemented for modern web browsers in javascript using the programming library `D3.js` (http://d3js.org/). The data generated by this study are to download along with the source code at https://github.com/andrewprayle/Do-guidelines-for-treating-chest-disease-in-children-use-Cochrane-reviews-effectively .

The original protocol used ordinary logisitic regression to examine the association between commissioning agency, publication year, topic and alternate high quality evidence upon the likelihood of citing a Cochrane Review. However, at the request of the statistical reviewer, we changed our analysis to a mixed effects logistic regression approach, to better account for the effect of clustering between guideline recommendations within guidelines.

We performed a series of mixed effects logistic regression models to study the association between of commissioning agency, publication year, topic and alternate high quality evidence upon the likelihood of citing a Cochrane Review. Due to the sparsity of data, and that several guidelines only contributed one recommendation to the dataset, we found that several of these models failed to converge using the **`glmer`** function in **`lme4`**. We found however that removing the 3 guidelines which contributed only one recommendation to the dataset allowed the model to converge when using the **bobyqa** optimization routine, and these results are reported below.

Supplementary results

Table S3 shows the guidelines included in the study and data collected.

Of the 96 recommendations that could use Cochrane Reviews, 29% (28/96) did not use any, and 10%, (10/96) did not use all the available Cochrane Reviews. There were 140 instances where a Cochrane Review could be linked to at least one guideline recommendation. Of these 103/140 (74%) were in agreement, 13/140 (9%) were partially in agreement, 5/140 (3.5%) disagreed and 19 / 140 (13%) were strong recommendations but the Cochrane Review did not draw a conclusion.

Table S4 shows the results of the sensitivity analysis. The original analysis suggests that 103/140 (74%) recommendations from respiratory guidelines in children are in line with the Cochrane Review. The figure remains the same if the 'partly in agreement' and 'not in agreement' categories are combined. However, if the 'partly in agreement' and 'totally in agreement' categories are combined, the agreement goes up to 116/140 (83%).

Association between commissioning agency, publication year, topic and alternate high quality evidence and use of Cochrane reviews

In this series of mixed effects logistic regression models, we found no evidence of an overall effect of commissioning agency (p = 0.99), publication year (p = 0.96), topic (p = 0.96) or alternate high quality evidence (p = 0.57). However, one commissioning agency (BTS / SIGN) was significantly less likely to cite Cochrane Reviews (odds ratio 0.24, 95% confidence interval 0.06 to 0.98, p=0.04). Table S5 shows the numbers of guidelines, recommendations, odds ratios and p values for these data.

In guidelines using the SIGN methodology for grading the evidence (n=7) only 53/289 (18%) of the recommendations were based on high quality evidence derived from meta-analyses, systematic reviews or randomised controlled trials with a low risk of bias. Approximately half of the guideline recommendations (133/289 or 46%) were based on case reports, case series, expert opinions or evidence extrapolated from case control or cohort studies, usually due to lack of availability of high quality evidence.

Further discussion

If a Cochrane review exists, is up to date, and is applicable to the guideline, we believe it should be cited in guideline recommendations.

Association between of topic, commissioning agency and use of Cochrane Reviews

Of the three largest groups (by number of recommendations in our study), asthma guidelines cited Cochrane Reviews the least, and respiratory infections and cystic fibrosis used the most. Part of this difference may be due to the amount of evidence available per topic. When fewer Cochrane Reviews are available, missing one will have a bigger effect on the proportion used. However, in the field of asthma there are multiple Cochrane Reviews which are relevant to asthma guidelines. The BTS / SIGN asthma guideline was significantly less likely to cite Cochrane Reviews. However, in this and other examples, any effect of the topic of the guideline could be confounded by commissioning agency and *vice versa*. We hypothesised that over time guidelines become more evidence based, and we examined whether publication year and use of other evidence affected Cochrane Review use. We found no evidence that the publication year or the use of other high quality was associated with Cochrane Review use.

Other work in the field

Silagy *et al*(1) looked at the use of Cochrane Reviews in clinical guidelines for the cessation of smoking and found four clinical practice guidelines, of which one was from the UK. In the UK guideline, Cochrane Reviews could have been used for 16/22 (73%) of the recommendations but were used for only half of these. This is in line with our finding that 60% of guideline recommendations for respiratory disease in children used all the relevant Cochrane Reviews. Brok *et al(2)* studied the agreement between guidelines and Cochrane Reviews for new-borns in Denmark. They found that 24% of guideline recommendations were not in agreement with the findings of a relevant Cochrane Review (of which 6% partially agreed, and 18% did not agree).

Other factors influencing strength of recommendations

Some of the discrepancy between the strength of recommendations and the strength of the evidence could be explained by other factors which should be taken into account when considering the strength of a recommendation. The current GRADE approach (3) proposes that recommendations are dichotomised into “strong” and “weak”. GRADE proposes four determinants of the strength of a recommendation: the quality of the evidence, the balance of risks and benefits, the variability in patient preference, and cost. It should be noted that not all the guidelines which we assessed used the GRADE methodology. When collecting data on alternate high quality evidence cited in guidelines, we did not assess the quality of this evidence with the GRADE approach; this is a topic for future work.

Commentary on the study methodology

Our study is comprehensive because all relevant clinical guidelines for respiratory disease written in the UK were included. We studied the Cochrane evidence base and national guidelines for the whole field of paediatric respiratory disease, at a single time-point. At the time of our search, it was surprisingly difficult to obtain all relevant guidelines, and some may have been overlooked when the main topic was not a respiratory disease or when the guideline was not indexed or tagged as a guideline or consensus document.

We strove for repeatability in our methodology by defining *a priori* what would constitute a relevant Cochrane Review, and defining agreement between guideline and Cochrane Review. The categorisation of agreement and linking Cochrane Reviews to guideline recommendations was done individually by two investigators, and we acknowledge the inherent subjectivity in this categorisation. Cochrane Reviews were only linked to guideline recommendations when the target group was the same, this however might under estimate the use of Cochrane Reviews in clinical guidelines. Guideline development takes time, and for this reason we pre-specified that Cochrane Reviews should be published at least one year prior to the publication of the guideline to for us to count them as “missed” if they were not cited. We found a small numbers of recommendations which were not in agreement with the conclusions of relevant Cochrane Reviews. Due to the low numbers in this group, commenting upon contributory factors would be speculative.

Explanations for non-citation of a Cochrane review

There may be legitimate reasons for not citing a Cochrane Review (such as the guideline development group not considering the intervention to be relevant or generalizable to the UK setting). Some guideline developers, such as the National Institute for Health and Care Excellence (NICE), commission their own systematic reviews to inform key recommendations and these are not published separately. The guideline group may not include a Cochrane Review if it has not been

updated at the specified interval. We excluded from our analysis Cochrane Reviews which had been withdrawn from the Cochrane Library. The Cochrane Review may cite only one relevant trial, in which case it is reasonable for the guideline to cite the trial rather than the review. However, in most guidelines, reasons for not citing Cochrane Reviews are not given and so it appears likely that high quality evidence is being overlooked.

There may be Cochrane Reviews which overlap (for example there are several Cochrane Reviews on corticosteroids in asthma). However, we believe that the guideline should consider all the available evidence, and this should be reflected in the citations within the guideline. There are no restrictions on number of citations in guidelines (as most are electronic) unlike journal articles (where the number of citations may be restricted). We would expect the experts in the field to be aware of all of the relevant Cochrane reviews, and this to be reflected in the guideline citations.

Limitations of this study

A key limitation of our study is that decisions on agreement of guideline recommendations with Cochrane Reviews were sometimes hard to achieve. We took a consensus approach where two investigators categorized, with independent adjudication of a third party. However, we recognise the subjectivity of this assessment. Decisions comparing 'partially in agreement' and 'totally in agreement' were particularly difficult. In the interests of transparency our raw data are available. In supplementary Table S1 we show the results of a sensitivity analysis in which we demonstrate the impact of changing our categorisations to group all the subgroups of 'not in agreement' into different categories. Additionally the interactive evidence network diagram allows easy visualisation and interrogation of our data.

We defined a Cochrane review as being relevant to a guideline recommendation on the basis of the same target group of patients, and that the Cochrane review could support or contradict the guideline recommendation. A limitation of this definition is that we did not consider whether the intervention was cost effective, feasible for the NHS to adopt and whether the intervention and setting was generalizable to the UK. This could lead to us overstating the number of Cochrane reviews which were potentially relevant to guideline recommendations.

Due to time and resource constraints, we had to limit our study to guideline recommendations for interventions. It is worth noting that there are large gaps in the evidence base for diagnosis and prognosis, and future studies should be directed at understanding these gaps in more detail.

It should be noted, that although Cochrane strives to update reviews, many reviews do become out of date. In a systematic analysis of the evidence base for interventions in paediatric primary care, only 44% of systematic reviews were up to date by the Cochrane collaboration's criteria.(4)

Although we systematically studied over 40 guidelines, of which 21 could be informed by Cochrane Reviews, which contained over one thousand recommendations, we found only and handful of recommendations which were (at least partially) at in disagreement with the conclusions of a relevant Cochrane Reviews. A larger study with a wider scope is required to study factors which make a guideline recommendation more likely to be at odds with the conclusions of what is often the best available evidence.   Additional work could also focus on conflict of interest and nationality of authors, and establish which guideline methodologies are associated with most reliably citing Cochrane Reviews.

Additional Tables and Figures

Table S1. Categorisation of agreement.

Category	*Discrepancies*	*Definition*
1	Totally in agreement	• Recommendations are the same<br>• There is weak evidence from a CR to support the recommendation in the guideline, and the guideline makes an appropriate recommendation<br>• Two interventions are equal to each other and the guideline promotes one for other non-efficacy/safety reasons (e.g. ease of administration, cost etc).
2	Partially in agreement	• Guideline makes a somewhat different recommendation than the CR.
3	Not in agreement	• Guideline makes a recommendation which is directly contradicted by the Cochrane Review.
4	Strong* guideline recommendation while there is no conclusion in the CR	• Guideline makes a strong recommendation while the CR concludes there is not enough evidence to make a recommendation.

A strong guideline recommendation was one in which there was a positive statement to do somehtine or not to do something, such as to administer a drug in a certain situation. An example of this would be to give corticosteroids for asthma at a certain dosage, or to administer an immunisation.

Table S2. Examples of categorisations.

Categorization type:	Guideline recommendation	Cochrane conclusion
1 Totally in agreement	In the absence of any evidence of benefit from the use of modified infant milk formulae it is not possible to recommend it as a strategy for preventing childhood asthma.	A large, well conducted trial of hydrolysed formula compared to cow's milk formula is required before hydrolysed formulas is offered routinely in preference to other types of formula …
2. Partially in agreement	The first choice as add-on therapy to inhaled steroids in adults and children is an inhaled long-acting beta-2 agonist which should be considered before going above a dose of 400 micrograms BDP or equivalent per day and certainly before going above 800 micrograms BDP (over 12s)	In adult patients who remain symptomatic on low dose inhaled steroids, the addition of a long-acting β2-agonist reduces the relative risk of exacerbations requiring systemic steroids by 17% as compared to that observed with the addition of a leukotriene receptor antagonist. […] The results may not be generalisable to children and adolescents, or patients over 65 years.
3. Not in agreement	If control remains inadequate on 400 micrograms daily of an inhaled steroid plus a long-acting beta-2 agonist consider increasing inhaled steroids to 800 micrograms BDP/day	Current asthma guidelines recommend titration of dose to individual patient response, but the published data provide little support for dose titration above 400 mcg/d in patients with mild to moderate asthma. There are insufficient data to draw any conclusions concerning dose-response in people with severe asthma.
4. Strong guideline but no conclusion in CR	Immunisations should be administered independent of any considerations related to asthma.	This review found very limited evidence to support the routine use of pneumococcal vaccine in people with asthma. A randomised trial of vaccine efficacy in children and adults with asthma is needed.

The first example of each class obtained in our data collection are shown here.

Table S3. Guidelines included in the study and data collected.

	Guideline	*Commissioner*	*Topic*	*Year*	*Recommendations made in guideline*				*Citation of relevant Cochrane Reviews in recommendations*		
					Total	*For treatment of lower respiratory disease in children*	*Recommendations for which a CR is available*	*Guideline recommendations which agree with all available CRs*	*All*	*Some*	*None*
1	British Guideline on the management of asthma	BTS, SIGN	Asthma	2009	146	75	31	20	11	7	13
2	Asthma (in children)- corticosteroids (TA131) (Inhaled corticosteroids for the treatment of chronic asthma in children under the age of 12 years)	NICE (TA)	Asthma	2007	4	4	3	0	3	0	0
3	Asthma (uncontrolled)-omalizumab (TA133)	NICE (TA)	Asthma	2007	6	5	0	NA	NA	NA	NA
4	Asthma (children under 5)-inhaler devices (TA10)	NICE (TA)	Asthma	2000	3	3	0	NA	NA	NA	NA
5	Asthma (older children)-inhaler devices(TA38)	NICE (TA)	Asthma	2002	5	5	0	NA	NA	NA	NA
6	Asthma (in children)-omalizumab (TA201) (Omalizumab for the treatment of severe persistent allergic asthma in children aged 6 to 11 years)	NICE (TA)	Asthma	2010	2	2	0	NA	NA	NA	NA
7	Methicillin-resistant staphylococcus aureus (MRSA)	CF Trust	Cystic fibrosis	2008	54	35	0	NA	NA	NA	NA
8	Standards of care and good clinical practice for the physiotherapy management of cystic fibrosis	CF Trust	Cystic fibrosis	2011	42	31	13	4	10	1	2
9	Antibiotic treatment for cystic fibrosis	CF Trust	Cystic fibrosis	2009	135	120	13	10	11	0	2
10	Nutritional management of Cystic Fibrosis	CF Trust	Cystic fibrosis	2002	47	33	1	0	0	0	1
11	Bronchiolitis in children a national clinical guideline	SIGN	Respiratory infections	2006	32	15	4	4	2	0	2
12	Tuberculosis: Clinical Diagnosis and Management of Tuberculosis and Measures for its Prevention and Control (117)	NICE (CG)	Respiratory infections	2011	153	71	1	1	0	0	1
13	Guidelines for non-CF bronchiectasis	BTS	Respiratory infections	2010	146	43	4	3	2	1	1
14	Recommendations for the assessment and management of cough in children	BTS	Respiratory infections	2007	13	13	5	4	3	1	1

15	Guidelines for the management of community acquired pneumonia in children	BTS	Respiratory infections	2011	33	16	4	4	4	0	0
16	Influenza-zanamivir, amantadine and oseltamivir (review) (TA168)	NICE (TA)	Respiratory infections	2009	5	4	2	2	1	0	1
17	Influenza (prophylaxis)-amantadine, oseltamivir and zanamivir (TA158)	NICE (TA)	Respiratory infections	2008	8	5	2	1	2	0	0
18	Pandemic flu: clinical management of patients with an influenza-like illness during an influenza pandemic	BTS, BIS, HPA, HD	Respiratory infections	2007	97	38	5	3	2	0	3
19	Respiratory tract infections (CG69)	NICE (CG)	Respiratory infections	2008	7	6	2	2	2	0	0
20	Standards for services for children with disorders of sleep physiology	RCPCH	Sleep apnoea	2009	26	11	1	0	1	0	0
21	A clinical guideline for the management of children presenting with acute breathing difficulty	RCPCH	Ventilation in peri-anaesthetic/critical care	2002	61	20	5	5	4	0	1
				Total	*1025*	*555*	96	*66*	*58*	*10*	*28*

Table S4. Results of sensitivity analysis.

	Number of recommendations				
	Totally in Agreement	Partially in agreement	Not in agreement	Strong guideline but no conclusion in CR	Overall agreement
Original analysis	103	13	5	19	103/140 (74%)
Case A – combine 'partially in agreement' with 'Not in agreement'	103	0	18	19	103/140 (74%)
Case B – combine 'Partially in agreement' with 'Totally in agreement'	116	0	5	19	116/140 (83%)

Table S5. Analysis of the influence of disease category and commissioning agency upon the likelihood of citing all relevant Cochrane Reviews.

Guideline parameter	Number of guidelines	Number of recommend-ations	Odds ratio of citing all the available Cochrane evidence	95% confidence interval	P value
**Disease category*1**					
Cystic Fibrosis	3	27	2.53	0.10 to 64.71	0.58
Respiratory infections	9	29	1.08	0.04 to 26.24	0.96
Ventilation in critical care	1	5	2.41	0.05 to 106.57	0.65
**Commissioning agency*2**					
BTS/BIS/HPA/DH	1	13	0.29	0.69 to 7.31	0.26
BTS/SIGN	1	31	0.24	0.06 to 0.98	0.04
CF Trust	3	27	1.86	0.40 to 8.61	0.42
NICE	5	10	3.55	0.32 to 38.78	0.30
RCPCH	2	6	1.77	0.14 to 21.40	0.65
SIGN	1	4	0.44	0.05 to 4.37	0.49

*1 – compared to baseline of asthma (which had 34 recommendations within 2 guidelines)
*2 – compared to baseline of BTS (which had 13 recommendations within 3 guidelines)
BTS = British Thoracic Society. SIGN = Scottish Intercollegiate Guidelines Network. BIS = British Infection Society. HPA = Health Protection Agency. NICE = National Institute for Clinical Excellence. CF Trust = Cystic Fibrosis Trust. RCPCH = Royal College of Paediatrics and Child Health. DH = Department of Health. P value calculated using the **glmer** function in R (using the option of setting the optimizer as the **bobyqa** algorithm and **nAGQ [**the number of points per axis for evaluating the adaptive Gauss-Hermite approximation to the log-likelihood**]** as 1[a Laplace optimisation]), comparing each category to the baseline.

REFERENCES

1. Silagy CA, Stead LF, Lancaster T. Use of systematic reviews in clinical practice guidelines: case study of smoking cessation. BMJ. 2001 Oct 13;323(7317):833-6. PubMed PMID: 11597966. Pubmed Central PMCID: 57801.
2. Brok J, Greisen G, Madsen LP, Tilma K, Faerk J, Borch K, et al. Agreement between Cochrane Neonatal reviews and clinical practice guidelines for newborns in Denmark: a cross-sectional study. Archives of disease in childhood Fetal and neonatal edition. 2008 May;93(3):F225-9. PubMed PMID: 17893123.
3. Guyatt GH, Oxman AD, Kunz R, Falck-Ytter Y, Vist GE, Liberati A, et al. Going from evidence to recommendations. BMJ. 2008 2008-05-08 22:01:04;336(7652):1049-51.
4. Gill PJ, Wang KY, Mant D, Hartling L, Heneghan C, Perera R, et al. The evidence base for interventions delivered to children in primary care: an overview of cochrane systematic reviews. PLoS One. 2011;6(8):e23051. PubMed PMID: 21829691. Pubmed Central PMCID: 3148227.

A systematic review of the use of Cochrane reviews in clinical guidelines, for lower respiratory disease, in children in the United Kingdom
Analysis plan

Objectives

1. To examine if all recommendations about interventions, relevant to respiratory diseases in Child Health, made in Cochrane reviews, are included in clinical practice guidelines for respiratory disease in Child Health in the UK.

2. To examine the cause when recommendations made in Cochrane reviews are not included in the guidelines or when the recommendations are only partially in agreement or in disagreement.

Guidelines

Search strategy

1. Guidelines will be searched in/on the website of:
   a. Embase
   b. Pubmed
   c. British Paediatric Respiratory Society
   d. British Thoracic Society (BTS)
   e. Cystic Fibrosis trust (CF Trust)
   f. Royal College of Paediatrics and Child Health (RCPCH)
   g. Scottish Intercollegiate Guidelines Network (SIGN)
   h. National Institute for Health and Clinical Excellence (NICE)
   i. Asthma UK
   j. British Lung Foundation
   k. Pulmonary Hypertension Association (PHA-UK)
   l. Alpha-1UK
   m. Breathtakers
   n. British Society for Allergy & Clinical Immunology
   o. British infection association

2. Search strategy for each database is as follows:

A	Embase*	paediatrics'/syn OR 'pediatrics'/syn OR 'child'/syn OR 'children'/syn OR infants OR new AND born OR 'new born' OR 'new borns' AND (respiratory OR 'lung'/syn OR 'respiration'/syn OR 'airway'/syn OR 'asthma'/syn OR 'pneumonia'/syn OR 'wheezing'/syn OR cf OR cystic AND 'fibrosis'/syn OR 'bronchiectasis'/syn OR 'bronchoconstriction'/syn OR 'coughing'/syn OR 'allergic rhinitis'/syn) AND ('uk'/syn OR united AND kingdom OR great AND 'britain'/syn OR british OR 'wales'/syn OR 'scotland'/syn OR 'england'/syn) AND [humans]/lim AND [1aediat]/lim AND (guideline OR 'consensus'/exp)
B	Pubmed*	(paediatrics[Title/Abstract] OR 1aediatrics[Title/Abstract] OR child[Title/Abstract] OR children[Title/Abstract] OR infants[Title/Abstract] OR (new[Title/Abstract] AND born[Title/Abstract]) OR new-born[Title/Abstract] OR new-borns[Title/Abstract]) AND (respiratory[Title/Abstract] OR lung[Title/Abstract] OR respiration[Title/Abstract] OR Airway[Title/Abstract] OR asthma[Title/Abstract] OR pneumonia[Title/Abstract] OR wheezing[Title/Abstract] OR CF[Title/Abstract] OR (cystic[Title/Abstract] AND fibrosis[Title/Abstract]) OR bronchiectasis[Title/Abstract] OR bronchoconstriction[Title/Abstract] OR coughing[Title/Abstract] OR

		wheezing[Title/Abstract] OR allergic rhinitis[Title/Abstract]) AND (UK[Title/Abstract] OR (United[Title/Abstract] AND Kingdom[Title/Abstract]) OR (Great[Title/Abstract] AND Britain[Title/Abstract]) OR British[Title/Abstract] OR Wales[Title/Abstract] OR Scotland[Title/Abstract] OR England[Title/Abstract]) AND "humans"[MeSH Terms] AND Practice Guideline[ptyp] AND English[lang] AND ("infant"[MeSH Terms] OR "child"[MeSH Terms] OR "adolescent"[MeSH Terms] AND (guideline OR consensus))
C	British Paediatric Respiratory Society	Guidelines
D	British Thoracic Society (BTS)	Guidelines
E	Cystic Fibrosis trust (CF Trust)	Consensus documents
F	Royal College of Paediatrics and Child Health (RCPCH)	Child health guidelines and standards: guidelines and standard developed by RCPCH, guidelines and standards developed by external organisations: endorsed by RCPCH: respiratory medicine, supported by RCPCH.
G	Scottish Intercollegiate Guidelines Network (SIGN)	Guidelines: child health, respiratory medicine
H	National Institute for Health and Clinical Excellence (NICE)	Filters:<br>i. Information type: guidance<br>ii. Conditions and diseases: respiratory<br>iii. Information type: all<br>iv. Public health: all<br>v. Treatments, procedures and devices: drug treatments, radiotherapy, surgical procedures, therapeutic procedures.<br>vi. Guidance type: all<br>vii. Publication status: all<br>viii. Date: all
I	Asthma UK	Guidelines/consensus documents
J	British Lung Foundation	Guidelines/consensus documents
K	Pulmonary Hypertension Association (PHA-UK)	Guidelines/consensus documents
L	Alpha-1UK	Guidelines/consensus documents
M	Breathtakers	Guidelines/consensus documents
N	British Society for Allergy & Clinical Immunology	BSACI guidelines
O	British infection association	Guidelines > Respiratory/Bacteria

*Search strategies for Pubmed and Embase are not interchangeable.

Selection strategy

1. Inclusion criteria for the guidelines will be:
    a. Clinical practice guidelines
    b. With at least one clear recommendation for an intervention
    c. For a respiratory disease including the lower airways up to and including anatomical sites of the epiglottis and including croup.
    d. For Child Health
    e. Written in the United Kingdom
    f. The last update
    g. Where the main topic is not diagnosing, screening, determination, investigation, testing for diagnosing, observations, or monitoring.
    h. When there is at least one Cochrane Review for the disease mentioned in the guideline title.

2. Exclusion criteria for the guidelines will be:
    a. Guidelines for cancer
    b. Guidelines about pregnancy

3. Inclusion criteria for the recommendations will be:
    a. Recommendations for children (0-18 years)
    b. About an intervention
    c. For a respiratory disease including the lower airways up to and including anatomical sites of the epiglottis and including croup.

4. Exclusion criteria for the recommendation will be, all recommendations about:
    a. Pregnancy
    b. Diagnosing
    c. Screening
    d. Determination
    e. Investigation
    f. Testing for diagnosing
    g. Observations or monitoring
    h. Recording
    i. Expert opinions
    j. Recommendations for specialists not directly affecting patients, e.g. hand washing protocols.

Data collection

1. Data collection from the guidelines:
    General data that will be collected from the guidelines includes the name of the guideline, the disease the guideline is for, information about: the guideline institution, funding, declaration of interest, publication date, update time, number of recommendations and the grading methodology used. This is specified in

Table 1: Guideline data collection in the appendix.

2. Data collection from the recommendations:
   Data that will be collected from the guideline recommendations includes the recommendations themselves, if they are included, and information about: the level of evidence used, patient's age, subcategory of patients/disease, moment of therapy, type of intervention, if the intervention is effective, and the date of literature search. This is specified in Table 2: Guideline recommendation data collection in the appendix.

Cochrane Reviews

Search strategy

1. Cochrane reviews will be searched in the:
   Cochrane Library

2. Search strategy will be*:
   a. Child Health
   b. Lungs and Airways

*We will not search the Cochrane "Ear Nose and Throat" topic. Croup and epiglottitis are dually listed under "Ear Nose and Throat" and "Respiratory infections" in the Cochrane Library.

Furthermore we will not include the Cochrane Library topic "Neonatal Care" in our search for Cochrane reviews. Our strategy will therefore miss than bronchopulmonary dysplasia (chronic lung disease) but we have not included a bronchopulmonary dysplasia guideline in our systematic review.

Selection strategy

1. Inclusion criteria for the Cochrane Reviews:
   a. Cochrane Reviews
   b. Further the same as for the guidelines. See Section: "Guidelines, selection strategy, 1b-g".

2. Inclusion criteria for the recommendations (author's conclusions) will be:
   a. Recommendations for children (0-18 years)
   b. For a respiratory disease including the lower airways up to and including anatomical sites of the epiglottis and including croup.

3. Exclusion criteria for the recommendations will be:
   a. The same as for the guidelines. See section: "Guidelines, selection strategy, 3, a-j". Further:
   b. Varicella
   c. Measles
   d. Meningitis
   e. Mumps
   f. Sinusitis
   g. Smoking cessation
   h. Sore throat

Data collection

1. Data collection from the Cochrane Reviews:

Data that will be collected from the Cochrane Reviews includes the Cochrane title, Cochrane number, the disease, the publication date, the number of recommendations and if the recommendation is clear. This is more specified in

Table 3: Cochrane Review data collection in the appendix.

2. Data collection from the recommendations:

   Data collected from the recommendations will include the recommendations themselves and information about: patient's age, subcategory of patients/disease, moment of therapy and if the intervention is effective. This is more specified in Table 4: Cochrane review recommendations data collection in the appendix.

Guidelines and Cochrane Review linked
Information from the guidelines and the Cochrane Reviews will be linked.

<u>Data collection</u>:
1. Per guideline relevant Cochrane reviews will be collected that could be cited and the Cochrane Reviews that are cited by the guideline. This is more specified in Table 5: Cochrane Reviews relevant to the guideline in the appendix.
2. Per guideline the recommendations will be linked to Cochrane recommendations if possible and discrepancies between them will be noted. This is more specified in Table 2: Guideline recommendation data collection in the appendix.

Outcomes
<u>Primary outcomes</u>
Guidelines:
1. Description of guideline recommendations:
   a. Mean/median number of recommendations
   b. Funder
   c. Year
   d. Declaration of interest
   e. Update time
   f. Guideline method
   g. Level of evidence used
   h. Type of interventions
2. Calculate the proportion of guideline recommendations which reference a Cochrane Review.
3. Calculate the proportion of guidelines and recommendations which do not refer to a Cochrane Review where a relevant review was identified.
   a. When the guideline did not cite the Cochrane Review:
      i. Refer the publication date of the guideline with the first publication date of the Cochrane Review
      ii. Guideline institution
      iii. Conflict of interest

Cochrane Reviews:
1. Description of Cochrane recommendations:
   a. Year
   b. Update time
   c. Type of interventions
   d. If the recommendations are clear

<u>Secondary outcomes</u>
1. Compare rates of referring to a Cochrane Review by:
   a. Conflict of interest
   b. Guideline institution

2. Calculate the proportion of Cochrane Reviews cited but which differ in recommendation.
    a. When there are discrepancies, refer number of discrepancies with:
        I. Guideline institution
        II. Level of evidence used (maybe another systematic review was used)
        III. Conflict of interest
3. Calculate the proportion of Cochrane Reviews not cited and which would have let to a different recommendation.

Appendix

Table 1: Guideline data collection 9
Table 2: Guideline recommendation data collection 11
Table 3: Cochrane Review data collection 20
Table 4: Cochrane review recommendations data collection 21
Table 5: Cochrane Reviews relevant to the guideline 22

Table 1: Guideline data collection

Guideline table	Arguments	Key
Guideline number	Numerical number to allocate the guideline.	Numerical number
Filenames	File name of the record.	e.g. “G0001.csv”
Guideline name	Identity of the guideline.	Copy the guideline name from the guideline
Guideline disease	Disease where the recommendations are made for.	Copy the disease
Name of the guideline institution	Guideline institution who wrote the guideline.	Copy the guideline institution’s name
Category of the guideline institution	Category institution who wrote the guideline.	0 = industry<br>1 = HTA<br>2 = NICE<br>3 = SIGN<br>4 = SIGN + other body<br>5 = professional body (e.g. RCPCH / BTS)<br>6 = NICE + other body<br>7 = other
Funding	Mentioned funder in the guideline	0 = unclear, not mentioned<br>1 = industry<br>2 = government<br>3 = foundation<br>4 = other<br>5 = not externally funded
Date of guideline publication	Date of guideline publications	Month and year of publication. E.g. “Dec-11”
Review date	Date the guideline is/will be reviewed	0 = unclear update time<br>Review date in time in years after first publication.
Update time mentioned in the guideline	Time period for the guideline to be updated given in the guideline.	0 = unclear update time<br>Update time in years.
Number of recommendations	Number of recommendations made in the guideline	Number of recommendations
Number of recommendations included	Number of recommendations made in the guideline which included in the research	Number of included recommendations
Methodology used in the guideline	Methodology used by the guideline.	Copy the used methodology
URL	For quick access to the guideline.	Copy the URL

		5 = easy; in the guideline
Completeness of declarations of interest	How complete the declarations of interest are. E.g. only mentioned that there is no conflict of interest or per person mentioned what the work functions are.	1 = not available<br>2 = unknown because difficult to access<br>3 = unspecified<br>4 = specified what conflicts are<br>5 = specified per person what all work functions are
Conflict of interest	If there is a conflict of interest	0 = unknown<br>1 = yes<br>2 = no
Grading	Methodology used for grading the evidence used in the guideline	0 = Unknown<br>1 = SIGN<br>2 = AHCPCR, 1992<br>3 = GRADE (Grading of Recommendations Assessment, Development and Evaluation system)<br>4 = HTA report<br>5 = Other
Level of evidence per recommendation known	If the level of evidence per recommendation is given in the guideline	0 = Inapplicable<br>1 = Yes<br>2 = No

Table 2: Guideline recommendation data collection

Guideline table	Arguments	Key
Guideline name	To identify the guideline.	Copy the guideline name from the master table of guidelines
Guideline number	To refer to the guideline	Copy the guideline number from the master table of guidelines
Filenames	File name of the record.	e.g. "0001.csv"
Number of recommendations	Number of recommendations made in the guideline.	Number of recommendations
Recommendation number	Numerical number to allocate the recommendation.	Numerical number
Page of recommendation	To easily find the recommendation	0 = inapplicable<br>Copy the page of recommendation
Paragraph of recommendation	To easily find the recommendation	Copy the paragraph of recommendation
Recommendation	Recommendation made in the guideline	Copy the recommendation
Recommendation included	e.g. recommendations about diagnosing are excluded	0 = included<br>1 = excluded
Level of evidence	Use SIGN methodology to determine the reliability of the recommendation.	0 = level of evidence unclear<br><br>**SIGN methodology**<br>1++ = High quality meta-analyses, systemic reviews of RCTs, or RCTs with a very low risk of bias.<br>1+ = Well conducted meta-analyses, systemic reviews, or RCTs with a low risk of bias.<br>1- = Meta-analyses, systemic reviews, or RCTs with a low risk of bias.<br>2++ = High quality systematic reviews of case control or cohort studies. High quality case control or cohort studies with a very low risk of confounding or bias and a high probability that the relationship is causal.<br>2+ = Well conducted case control or cohort studies with a low risk of confounding or bias and a moderate probability that the relationship is causal.

2- =	Case control or cohort studies with a high risk of confounding or bias and a significant risk that the relationship is not causal
3 =	Non-analytical studies, eg case reports, case series
4 =	Expert opinion

Converting GRADE methodology to SIGN methodology:

QoE (quality of evidence) GRADE methodology	*Converted to SIGN methodology*
High	1
Moderate	2
Low	3
Very low	4

Converting AHCPR methodology to SIGN methodology:

Level of evidence AHCPR methodology	*Converted to SIGN methodology*
A: Requires at least one randomised controlled trial as part of the body of literature of overall good quality and consistency addressing the specific recommendation.	1 | 2++
B: Requires availability of well conducted clinical studies but no randomised clinical trials on the topic of the recommendation.	2 | 3
C: Requires evidence from expert committee reports or opinions and/or clinical experience of respected authorities. Indicates absence of directly applicable studies of good quality.	3 | 4

Converting level of evidence used in the BTS guideline: Guidelines for the management of community acquired pneumonia in children, to SIGN methodology:

Level of evidence BTS methodology	*Converted to SIGN methodology*
A+: A good recent systematic review of studies designed to answer the question of interest	1 | 2
A-: One or more rigorous studies designed to answer the question, but not formally combined	2
B+: One or more prospective clinical studies which illuminate, but do not rigourisly answer, the question	2
B-: One or more retrospective clinical studies which illuminate, but do not rigoursly answer, the question	2
C: formal combination of expert views	4
D: other information	4

<table>
<tr><td></td><td></td><td>Converting BTS methodology to SIGN methodology when using SIGN methodology but only given A/B/C/D: (guidelines for non-CF bronchiectasis / RCPCH: standards for services for children with disorders of sleep/ RCPCH: A clinical guideline for the management of children presenting with acute breathing difficulty

<table>
<tr><td>Level of evidence BTS methodology</td><td>Converted to SIGN methodology</td></tr>
<tr><td>A</td><td>1 ++ | 1+</td></tr>
<tr><td>B</td><td>1 | 2</td></tr>
<tr><td>C</td><td>2</td></tr>
<tr><td>D</td><td>3 | 4</td></tr>
</table>

Converting level of evidence of the BTS guideline: management of opportunist mycobacterical infections, to SIGN methodology:

<table>
<tr><td>Level of evidence BTS methodology</td><td>Converted to SIGN methodology</td></tr>
<tr><td>A: Requires at least one randomised controlled trial as part of the body of literature of overall good quality and consistency addressing the specific recommendation.</td><td>1 | 2++</td></tr>
<tr><td>B: Requires availability of well conducted clinical studies but no randomised clinical trials on the topic of recommendation.</td><td>2</td></tr>
<tr><td>C: Requires evidence from expert committee reports or opinions and/or clinical experience of respected authorities, but indicates absence of directly applicable studies of good quality.</td><td>3 | 4</td></tr>
</table>

X = recommendation excluded</td></tr>
<tr><td>Decision for the level of evidence</td><td>Who specified the level of evidence</td><td>0 = level of evidence specified by the guideline
1 = level of evidence specified by us
2 = level of evidence unclear

X = recommendation excluded</td></tr>
<tr><td>Grades of recommendations</td><td>Use SIGN methodology to determine the reliability of the recommendation.</td><td>0 = grade of recommendation unclear

SIGN methodology
A = At least one meta-analysis, systematic review, or RCT rated as 1++,and directly applicable to the target population; or A body of evidence consisting principally of studies rated as 1+,directly applicable to the target population, and demonstrating overall consistency of results.</td></tr>
</table>

		B = A body of evidence including studies rated as 2++, directly applicable to the target population, and demonstrating overall consistency of results; *or* Extrapolated evidence from studies rated as 1++ or 1+<br>C = A body of evidence including studies rated as 2+,directly applicable to the target population and demonstrating overall consistency of results; *or* Extrapolated evidence from studies rated as 2++.<br>D = Evidence level 3 or 4; or Extrapolated evidence from studies rated as 2+<br><br>**Converting GRADE methodology to SIGN methodology:**<br>*QoE (quality of evidence) GRADE methodology* — *Converted to SIGN methodology*<br>High — A/B<br>Moderate — C<br>Low — D<br>Very low — D<br><br>**Converting AHCPR methodology to SIGN methodology:**<br>*Level of evidence AHCPR methodology* — *Converted to SIGN methodology*<br>A — A | B<br>B — B | C<br>C — D<br><br>**Converting BTS methodology to SIGN methodology:**<br>*Level of evidence BTS methodology* — *Converted to SIGN methodology*<br>A+: A good recent systematic review of studies designed to answer the question of interest — A | B<br>A-: One or more rigorous studies designed to answer the question, but not formally combined — B | C<br>B+: One or more prospective clinical studies which illuminate, but do not rigourisly answer, the question — B | C<br>B-: One or more retrospective clinical studies which illuminate, but do not rigourly answer, the question — B | C<br>C: formal combination of expert views — D<br>D: other information — D

<table>
<tr><td></td><td></td><td>Converting level of evidence of the BTS guideline: management of opportunist mycobacterical infections, to SIGN methodology:
<table><tr><td>Level of evidence BTS methodology</td><td>Converted to SIGN methodology</td></tr><tr><td>A: Requires at least one randomised controlled trial as part of the body of literature of overall good quality and consistency addressing the specific recommendation.</td><td>A | B</td></tr><tr><td>B: Requires availability of well conducted clinical studies but no randomised clinical trials on the topic of recommendation.</td><td>B | C</td></tr><tr><td>C: Requires evidence from expert committee reports or opinions and/or clinical experience of respected authorities, but indicates absence of directly applicable studies of good quality.</td><td>D</td></tr></table>
X = recommendation excluded</td></tr>
<tr><td>Decision for the grades of recommendation</td><td>Who specified the level of evidence</td><td>0 = grade of recommendation specified by the guideline
1 = grade of recommendation specified by us
2 = grade of recommendation unclear

X = recommendation excluded</td></tr>
<tr><td>Patient's age</td><td>Age of the patients the recommendation is made for.</td><td>0= inapplicable
Age in years e.g. 4 – 8.5

Baby: 0-1
Neonate: 0- 1/12

X = recommendation excluded</td></tr>
<tr><td>Subcategory of patients/disease</td><td>Category of patients/disease. E.g. Acute asthma versus chronicle asthma.</td><td>0 = inapplicable
1 = pregnant
2 = smoking
3 = symptomatic
4 = mild
5 = moderate
6 = severe
7 = life threatening
8 = breastfeeding
9 = other
10 = acute
11= chronic</td></tr>
</table>

		12 = parents<br><br>X = recommendation excluded
Moment of therapy	At which moment of therapy the intervention starts.	0 = inapplicable<br>1 = starting<br>2= stepping up<br>3 = maintenance<br>4 = stepping down<br>5 = exacerbations<br>6 = continue<br>7 = add on<br><br>X = recommendation excluded
Type of intervention	Type of intervention. E.g. physiotherapy.	0 = avoidance<br>1 = nutritional/herbal<br>2 = exercise/physiotherapy<br>3 = prevention<br>4 = pharmacological<br>5 = surgical<br>6 = weight reduction<br>7 = quit smoking<br>8 = other<br>9 = supporting tool<br>10 = referral to specialist<br>11 = educational/informational<br>12 = psychotherapy<br>13 = hospitalisation<br>14 = type of device<br><br>X = recommendation excluded
Effective intervention	If an intervention is effective or not	0 = effective/recommended<br>1 = not effective/ not recommended<br>2 = intervention not more or less favoured over one other/insufficient evidence to make a choice<br><br>X = recommendation excluded
Specific medicine	Medicine recommended	0= inapplicable<br>Copy the name of the medicine recommended

		X = recommendation excluded
Main group of the medicine	Main group of the recommended medicine	0= inapplicable<br><br>Copy the name and number of the main group of the medicine from the table. If the medicine is not mentioned in the table: put the name and number of the main group (See BNF for children) in the table and copy from the table.

Main group of the medicine	*Number of the main group of the medicine*
Allergen immunotherapy	3.4.2
Allergic emergencies	3.4.3
Aminoglycosides	5.1.4
Anaemias and some other blood disorders	9.1
Antibacterial drugs	5.1
Antifibrinolytic drugs and haemostatics	2.11
Antifungal drugs	5.2
Antihistamines	3.4.1
Antimuscarinic bronchodilators	3.1.2
Antituberculosis drugs	5.1.9
Antiviral drugs	5.3
Bronchodilators	3.1
Cephalosporins, carbapenems, and other beta-lactams	5.1.2
Compound bronchodilator preparations	3.1.4
Corticosteroids	6.3
Cough suppresants	3.9.1
Cromoglicate and related therapy	3.3.1
Drugs used in nasal allergy	12.2.1
Immunoglobulins	14.5
Inhaled corticosteroids	3.2
Leukotriene receptor antagonists	3.3.2
Macrolides	5.1.5
Magnesium	9.5.1.3
Minerals	9.5
Mucolytics	3.7
Nasal preparations for infection	12.2.3
Non-steroidal anti-inflammatory drugs	10.1.1
Oral glucocorticoid therapy	6.3.2
Oxygen	3.6

<table>
<tr><td></td><td></td><td><table>
<tr><td>Penicillins</td><td>5.1.1</td></tr>
<tr><td>Proton pump inhibitors</td><td>1.3.5</td></tr>
<tr><td>Quinolones</td><td>5.1.12</td></tr>
<tr><td>Selective beta 2 agonists</td><td>3.1.1.1</td></tr>
<tr><td>Skin cleansers, antiseptics, and preparations for promotion of wound healing</td><td>13.11</td></tr>
<tr><td>Some other antibacterials</td><td>5.1.7</td></tr>
<tr><td>Sulphonamides and trimethoprim</td><td>5.1.8</td></tr>
<tr><td>Tetracyclines</td><td>5.1.3</td></tr>
<tr><td>Theophylline</td><td>3.1.3</td></tr>
<tr><td>Topical nasal decongestants</td><td>12.2.2</td></tr>
<tr><td>Vaccines and antisera</td><td>14.4</td></tr>
<tr><td>Vitamins</td><td>9.6</td></tr>
</table>
X = recommendation excluded</td></tr>
<tr><td>Number of the main group of the medicine</td><td>The main group recommended for intervention can be the same while the subgroup might differ.</td><td>0= inapplicable

Copy the name and number of the main group of the medicine from the table (table can be found at main group of the medicine). If the medicine is not mentioned in the table: put the name and number of the main group (See BNF for children) in the table and copy from the table.

X = recommendation excluded</td></tr>
<tr><td>Dosage</td><td>Total dose/day of the medicine for a stated amount of weight</td><td>0= inapplicable
Copy the total dose (mg)/day for a child of 20 kg's.

X = recommendation excluded</td></tr>
<tr><td>Date of literature search</td><td>A guideline might have different dates of literature search for different chapters.</td><td>0 = date not clear
Copy the date of literature search per recommendation.

X = recommendation excluded</td></tr>
<tr><td>Title of Cochrane Review used</td><td>If a Cochrane review was used for the recommendation</td><td>Title(s) of the used Cochrane reviews

X = recommendation excluded</td></tr>
<tr><td>Number of Cochrane Review used</td><td>,,</td><td>Number(s) of the title(s) of the Cochrane reviews.

X = recommendation excluded</td></tr>
</table>

Agreement between review and guideline	Agreement between the recommendations in the Cochrane review and the guideline.	0 = inapplicable<br>1 = completely/ No evidence in CR but the guideline has to make a recommendation and takes in mind the fifferent target group, adverse effects, pharmacovigilance, costs etc.<br>2 = partial<br>3 = no agreement<br>4 = No evidence and guidelines makes a strong recommendation<br><br>Give examples<br><br><br>X = recommendation excluded
Title of Cochrane Review could be used	Cochrane review relevant to the guideline	Title(s) of the Cochrane reviews that could be used<br><br>X = recommendation excluded
Number of Cochrane Review could be used	Cochrane review relevant to the guideline	Number(s) of Cochrane reviews that could be used.<br><br>X = recommendation excluded
Comment	Place to make a comment is there is something extraordinary	Type the comment<br><br>X = recommendation excluded
Recommendation included	If the recommendation in the guideline is included	0 = included<br>1 = excluded
Who found the Cochrane Review	If the Cochrane Review was included in the guideline or not	0 = inapplicable/recommendation excluded<br>1 = Guideline linked the Cochrane Review to the recommendation as well (Both).<br>2 = Guideline linked the Cochrane Review to the recommendation but we did not (They).<br>3 = We linked the Cochrane Review to the recommendation but the guideline did not (We).

Table 3: Cochrane Review data collection

**Cochrane Review table**	**Arguments**	**Key**
Cochrane number	Numerical number to allocate the Cochrane Review.	Numerical number
Filename	File name of the record.	e.g. “C0001.csv”
Cochrane title	Identity of the Cochrane Review.	Copy the Cochrane Review name from the Cochrane Review.
Cochrane disease	Disease where the recommendations are made for.	Copy the heading used in the Cochrane Library
Date of publication	Date of Cochrane Review publications	Month and year of publication. E.g. “Dec-11”
Date of first publication	Publication date of the first version of the Cochrane Review	Copy the date of “review first published” in the Cochrane Review under “history”.
Number of recommendations	Number of recommendations made in the Cochrane Review.	Number of recommendations
Clear recommendation	If the recommendation is clear or not	0 = (no recommendation possible as) no evaluable evidence. Might be a clear recommendation but there is no evidence to confirm (e.g. C0282.csv)<br>1 = “soft”/partial recommendation: recommendation made but more evidence necessary to confirm or when “may” is used in the recommendation/effective but benefits have to be weighed against the risk of side effects/clear recommendation but more evidence necessary to confirm.<br>2 = clear neutral recommendations: no significance difference between interventions<br>3 = clear positive recommendation<br>4 = clear negative recommendation<br>5 = clear recommendation (nor positive nor negative)
URL	For quick access to the Cochrane Review.	Copy the URL
Comment	Place for making a comment if necessary	Type the comment
Accessed date	Accessed date of the Cochrane Review	Accessed date of the Cochrane Review. “e.g. 03/07/2012”

Table 4: Cochrane review recommendations data collection

Cochrane Review table	Arguments	Key
Cochrane title	To identify the Cochrane Review.	Copy the Cochrane Review name from the master table of Cochrane reviews.
Cochrane number	To refer to the Cochrane Review	Copy the Cochrane Review number from the master table of Cochrane Reviews
Filename	File name of the record.	e.g. "C0001.csv"
Number of recommendations	Number of recommendations made in the Cochrane Review.	Number of recommendations
Recommendation number	Numerical number to allocate the recommendation.	Numerical number
Recommendation	Recommendation made in the Cochrane Review	Copy the author's conclusions form the Cochrane Review
Recommendation included	See Table 2: Guideline recommendation data collection	
Patient's age		
Subcategory of patients/disease		
Moment of therapy		
Type of intervention		
Specific medicine		
Main group of the medicine		
Number of the main group of the medicine		
Dosage		

Table 5: Cochrane Reviews relevant to the guideline

Cochrane Reviews and Guidelines linking table	Arguments	Key
Name of guideline	To identify the guideline.	Copy the guideline name from the master table of guidelines
Number of guideline	To refer to the guideline	Copy the guideline number from the master table of guidelines
File name	File name of the record.	e.g. “L0001.csv”
Number	Number of the inserted data	e.g. “ 1”
Title of Cochrane Review referred to in the guideline	Title of Cochrane Review mentioned in the references	Copy the title of the Cochrane Review<br>0 =inapplicable
Number of Cochrane Review referred to in the guideline	Number of Cochrane Review mentioned in the references	Copy the number of the Cochrane Review<br>0 =inapplicable
Comment	Reason for excluding a Cochrane Review/ we did not find the Cochrane Review	If applicable type the comment<br>0 =inapplicable
Cochrane Review linked to a recommendation by the guideline	If a Cochrane Review is in agreement with a specific recommendation made in the guideline	0 = inapplicable (Cochrane Review excluded/withdrawn from publication)<br>1 = yes<br>2 = no
Title of Cochrane Review found by us	If a Cochrane Review is relevant to a guideline on basis of the name of the guideline or of a recommendation made in the guideline	Copy the title of the Cochrane Review<br>0 =inapplicable
Number of Cochrane Review found by us	If a Cochrane Review is relevant to a guideline on basis of the name of the guideline or of a recommendation made in the guideline	Copy the number of the Cochrane Review<br>0 =inapplicable
Cochrane Review linked to a recommendation by us	If a Cochrane Review is relevant to a recommendation in the guideline based on that recommendation	0 = inapplicable (did not find the Cochrane Review)<br>1 = yes<br>2 = no
Who found the Cochrane Review	If the Cochrane Review is cited by the guideline or not.	0 = inapplicable<br>1 = the guideline as well<br>2 = only the guideline<br>3 = only we<br>4 = only the guideline but excluded. E.g. pregnancy/adults only/withdrawn
